# Lower versus higher frequency of sessions in starting outpatient mental health care and the risk of a chronic course; a naturalistic cohort study

**DOI:** 10.1186/s12888-019-2214-4

**Published:** 2019-07-24

**Authors:** Bea Tiemens, Margot Kloos, Jan Spijker, Theo Ingenhoven, Mirjam Kampman, Gert-Jan Hendriks

**Affiliations:** 1 Pro Persona Research, Renkum, The Netherlands; 2 Behavioural Science Institute, Radboud University, Nijmegen, The Netherlands; 3 Depression Expertise Centre, Pro Persona Mental Health Care, Nijmegen, The Netherlands; 4 Personality disorder Expert Centre, Arkin Mental Health Care, Amsterdam, The Netherlands; 5 Overwaal Centre of Expertise for Anxiety Disorders, OCD and PTSD, Pro Persona Mental Health Care, Nijmegen, The Netherlands; 6 Department of Psychiatry, Radboud University Medical Centre, Nijmegen, The Netherlands

**Keywords:** Session frequency, Depression, Anxiety, Personality disorder, Chronicity

## Abstract

**Background:**

An adequate frequency of treatment might be a prerequisite for a favorable outcome. Unfortunately, there is a diversity of factors that interfere with an adequate frequency of sessions. This occurs especially in the first phase of treatment, while the first phase seems vital for the rest of treatment. The aim of this naturalistic study was to explore the impact of the initial frequency of treatment sessions on treatment outcome in a diverse mental health care population.

**Methods:**

Anonymized data were analyzed from 2,634 patients allocated for anxiety disorders, depressive disorders, and personality disorders to outpatient treatment programs in a large general mental health care facility. Patients’ treatment outcome was routinely monitored with the Outcome Questionnaire-45 (OQ-45.2), every 12 weeks. Frequency of sessions was assessed for the first three months of treatment. Using Cox-proportional-hazard models, we explored the associations between initial frequency and improvement (reliable significant change) and recovery (reliable and clinically significant change).

**Results:**

Improvement and recovery were associated with symptom severity and functional impairment at start of treatment, the year the treatment started, number of measurements, the treatment program (anxiety disorders, depressive disorders, and personality disorders) and receiving group therapy other than psychotherapy.

In all diagnostic groups, both improvement and recovery were associated with a higher frequency of sessions during the first three months of treatment. For improvement, this effect diminished after three years in treatment; however, for recovery this association was sustained.

**Conclusions:**

In addition to severity at start of treatment and other predictors of outcome, a low frequency of initial treatment sessions might lead to a less favorable outcome and a more chronic course of the mental disorder. This association seems not to be limited to a specific diagnostic group, but was found in a large group of patients with common mental disorders (depression and anxiety disorders) and patients with a personality disorder. Despite organizational obstacles, more effort should be made to start treatment quickly by an effective frequency of session.

## Background

In the last decades, remarkable progress has been made in outpatient mental health care with the introduction of evidence-based, clinical guidelines for mental disorders. Nevertheless, in everyday practice, a large number of patients do not benefit from their treatment [1]. Despite having received treatment, a considerable proportion of patients suffer from their symptoms for a very long time and develop persisting symptoms and impairments, for instance in functioning in their social roles [2–4]. On the one hand, it has often been suggested that differences in treatment outcome between mental health care practice and randomized controlled trials (RCT) are attributable to the different populations, but this has not been empirically confirmed [5]. On the other hand, reasons for responding or not responding to treatment are generally sought in patient characteristics. However, even the most obvious patient characteristics are not unequivocally predictive of treatment outcome. For example, for depression or anxiety sometimes gender is a predictor of treatment outcome [6], but often not [7, 8]. Sometimes age seems a predictor [7], but often not [6, 8]. For both depression and anxiety, functional impairment or (absence of) work often is a predictor [7–9], just like having a comorbid personality disorder [6, 8, 10]. Severity of symptoms at the start of treatment is supposed to be an important predictor of treatment outcome as found for social anxiety [11], depression [9] and adjustment disorders [12], but not in a very large individual patient data meta-analysis for depression [13]. In the current study, we addressed another, often neglected, potential reason for the difference in treatment outcome between mental health care practice and randomized controlled trials, viz., a low dose of the initial treatment.

Dose-effect models of psychotherapy, as described in the 1980s, showed that most improvement occurs during the initial stage of treatment [14]. However, these models depict an average improvement curve, and do not distinguish between different patterns of change as was shown in Barkham et al.’s [7] good-enough-level model. These authors found many variations in change patterns, especially in the early phase of treatment. This finding was confirmed by Rubel et al. [15] who found that the largest variation in change patterns occurred during the first six sessions of outpatient psychotherapy. The largest improvement, as in the dose-effect model, was found in the first phase of treatment. In later phases, i.e., sessions 7–12 and 13–18, the majority of patients showed either a small improvement, or no change at all. Moreover, the patients whose problems deteriorated in the first phase of treatment generally did not improve substantially in the latter phases. The studies of Barkham et al. and Rubel et al. included patients who received outpatient treatment for a variety of problems, but these were mainly common mental disorders such as depression or anxiety disorders. However, similar patterns of change were found in the treatment of patients with personality disorders not otherwise specified, regardless of the treatment modality being used [16]. Improvement was greatest in the first 12 months of treatment.

The focus on these patterns of change in the first phase of treatment is relevant because a patient’s early change pattern seems to be a predictor of the final outcome of treatment. In different patient groups and settings, early treatment response has been found to be strongly related to a positive outcome [17–19]. This predictive indicator of treatment response might even occur very soon after the start of treatment, e.g., within the first month or even after only a few sessions [12, 20].

In studies showing the variation in and predictive power of early change in outpatient treatment, the first phase of treatment was usually defined in terms of either the first three-to-six sessions or some other specific initial time period. According to most evidence-based protocols, weekly sessions are recommended, e.g., five sessions is similar to five weeks or eight weeks refers to eight sessions. However, in routine mental health care, weekly sessions often are not possible because of waiting lists and scheduling (planning) difficulties in mental health care agencies resulting from patients’ or therapists’ vacations and sick leaves or patients’ other practical difficulties. The importance of the frequency of sessions might also be underestimated, even though in a meta-regression analysis Cuijpers et al. [21] confirmed the importance of the frequency of sessions. They found among patients being treated for depression a very strong association between the number of sessions per week and the treatment effect size. On the other hand, the total number of sessions and the duration of the therapy were hardly or not at all associated with treatment outcome, but increasing the frequency of the sessions from one per week to two per week increased the effect size by .45. There is some evidence among patients with a social anxiety disorder that less intensive treatment is less effective than therapy delivered in weekly sessions [22]. Erekson et al. found similar results in the case of psychotherapy delivered in a naturalistic setting to students with a variety of psychological problems, mainly adjustment, anxiety, or depression related [23]. Additionally, Omar et al. conducted a systematic review of studies with patients with borderline personality disorder [24]. They reported better outcomes in terms of self-harm, depression, and social functioning when patients received more than one individual session per week or when patients received group-based sessions.

In summary, the following evidence served as the backdrop of the present study: (a) the largest improvement in symptoms occurs in the first phase of treatment, (b) during later phases of treatment there is little additional change in symptoms, (c) improvement or lack of improvement in the first phase of treatment is associated with final treatment outcome, and (d) the frequency of sessions is associated with final outcome. We were interested in identifying the association between the rapidity with which therapy starts (in terms of frequency of sessions) and the speed of recovery. This study was designed to answer two specific questions: If a low frequency of sessions in the first phase of treatment leads to an unfavorable pattern of change in this phase, is this pattern associated with both slow recovery in the following phases of treatment and even with a chronic course of the illness? If there is an overall association between speed of initial treatment and the speed of recovery, is this pattern similar among patients with depression, an anxiety disorder, or a personality disorder? Most of the studies mentioned so far mainly concerned patients with depression or an anxiety disorder. The current study also included data from patients with a personality disorder because of the high comorbidity with depression and anxiety and because some studies have shown that the presence of a personality disorder has a negative effect on the outcome of depression or an anxiety disorder [6, 8, 10]. In fact the question of the present study is, can some more general pattern of change be identified that should be taken into account during treatment planning?

In the present study, we analyzed data from Routine Outcome Monitoring (ROM) of patients being treated in the outpatient treatment programs for a depressive disorder, an anxiety disorder, an obsessive-compulsive disorders, or a personality disorder in a large mental health care institution in the east of the Netherlands. In this naturalistic study in which we used data from patients who started treatment during five consecutive years, we expected to find an association between frequency of sessions in the first phase of treatment, the speed of recovery, and the final treatment outcome.

## Methods

### Design

The aim of the study was to test our hypothesis about the association between the frequency of sessions in the first phase of treatment and the speed of recovery and final outcome. Therefore, we conducted a retrospective cohort study in a naturalistic setting for three broad diagnostic groups: outpatients who were allocated to treatment for a depressive disorder, an anxiety disorder (including obsessive-compulsive disorder and post-traumatic stress disorder), or a personality disorder.

### Patient population and procedure

Data were collected in a large mental health care facility in the eastern part of the Netherlands. This facility offers treatment that is reimbursed by basic health care insurance. Requirements for reimbursement were that (a) the patient had been referred by his or her general practitioner and (b) the patient’s symptoms met the criteria for a DSM-IV diagnosis (or a DSM-5 diagnosis, which was implemented in the Netherlands in 2017). During each patient’s intake, the diagnosis was assessed and confirmed in a semi-structured clinical interview (Dutch version of M.I.N.I. 5.0.0 [25] and SCID-II). Patients were, depending on their primary DSM-IV diagnosis, allocated to one of the specialized treatment programs. These programs are based on the evidence-based Dutch multidisciplinary guidelines for mental health care (https://www.ggzrichtlijnen.nl). In this study, data were included from outpatients treated for a depressive disorder, an anxiety disorder (including obsessive-compulsive disorder and post-traumatic stress disorder), or a personality disorder. Treatment consisted of psychotherapy with or without medication. Most patients received individual therapy, but a small proportion of the patients received group therapy. Each of the therapists was a qualified psychotherapist, psychologist, psychiatrist, or psychiatric nurse.

Data were obtained from the electronic registration system and from routine outcome monitoring (ROM), which is a part of usual care. Patients were routinely asked to fill out the Outcome Questionnaire-45 (OQ-45.2) at the registration for treatment, at the onset of treatment, after every three months (12 weeks), and at the end of treatment. At each assessment point, patients received an e-mail message from the electronic ROM system with a link to the questionnaire. Patients for whom an e-mail address was not available received a letter with a description of the procedure for logging into the ROM system and filling out OQ-45.2. Patients who did not have access to a computer or Internet connection were offered the opportunity to fill out the paper version of the OQ-45.2 or to use one of the special ROM computers at the location where they received their treatment.

Data from patients who started treatment in 2011, 2012, 2013, 2014, or 2015 was included in the study. In order for the sample to resemble the usual patient population, no exclusion criteria were used.

Ethical approval for conducting the study was not required because the measures and assessment procedure were part of usual care and anonymized data were used. Information which does not relate to an identified or identifiable natural person does not fall under the European General Data Protection Regulation. At the intake all patients of the mental health care facility were informed about the organization’s policy on the use of anonymized data for improvement of the quality of care through scientific research. Patients then had the opportunity to decline having their anonymized data used for scientific research. The data of patients who had declined were removed from the dataset.

### Definition of session-frequency categories

Frequency of sessions was assessed during the first three months of treatment. Because, patients filled out the OQ-45 every three months, unless the treatment was terminated earlier, this was the first possibility to measure improvement or recovery.

The number of sessions was counted from the start of the treatment in the respective treatment programs. To achieve uniformity in the study period in the three programs, we excluded the intake session or other contacts before the actual treatment started. When waiting lists were long, sometimes a patient had contacts with a therapist during the waiting period, but these were not counted as prescribed treatment contacts. In the three months from the start of the actual treatment, all face-to-face treatment contacts (both psychotherapy and pharmacotherapy contacts) were counted.

In order to be able to distinguish between different frequency conditions in the Kaplan Meier survival curves, we divided the number of sessions in the first three months into categories. We found a large variation in the number of sessions, which ranged from 1 to 65 (mean = 9.3, sd = 7.7; see Table 1). We constructed five categories: 1 to 3 sessions (i.e., a maximum of one session per month); 4 to 6 sessions (a maximum of two sessions per month); 7 to 9 sessions; 9 to 12 sessions (a maximum of one each week); and more than 12 sessions (more than one session per week).

**Table 1:** Patient characteristics and potential confounders

Characteristic	Categories	N (%) or Mean (SD) *N* = 2,634
Sex	Male	981 (37.2%)
	Female	1653 (62.8%)
Age (in years)		36.1 (12.0)
Year treatment started	2011	429 (16.3%)
	2012	765 (29.0%)
	2013	871 (33.1%)
	2014	446 (16.9%)
	2015	123 (4.7%)
Diagnostic program	Anxiety	1238 (47.0%)
	Depression	922 (35.0%)
	Personality disorders	474 (18.0%)
Number of sessions in the first 3 months	*Range from 1 to 64*	9.3 (7.7)
	1–3	421 (16.0%)
	4–6	650 (24.7%)
	7–9	324 (12.3%)
	10–12	701 (26.6%)
	> 12	538 (20.4%)
At least 1 pharmacotherapy contact		774 (29,4%)
Number of available measurements		4.2 (2.4)
Group treatment	No group treatment	1942 (73.7%)
	Psychotherapy group	157 (6.0%)
	Another kind of group	535 (20.3%)
OQ-45 total score at start		87.4 (23.4)
GAF score at start		54.9 (8.1)
Improved		1149 (43.6%)
Recovered		649 (24.6%)

### Outcome measures

Treatment outcome was measured with the Outcome Questionnaire-45 (OQ-45.2) [26] [27], which is a self-report questionnaire for general psychopathology and functioning. The OQ-45.2 is designed for repeated measurement of clients’ progress during therapy in three domains: symptom distress (SD), interpersonal relationships (IR), and social role (SR). Each of the 45 items is answered on a five-point Likert scale ranging from *never* (a score of *0*) to *almost always* (score of *4*), according to the patient’s recollection of the preceding week. An overall total score is calculated by adding the item scores. The total score can range from *0* to *180*. Higher scores reflect more severe distress; a decrease in the total score indicates improvement. The Dutch translation of the OQ-45.2 was used; it has satisfactory psychometric properties [27]. The Dutch OQ discriminates between functional and dysfunctional populations and the concurrent validity showed proper values for the total score and the SD subscale, but was moderate for the IR and SR subscales. The OQ showed high sensitivity to change on all subscales, ranging from Cohen’s d = 0.77 to 1.33 (total score). Test-retest reliability was in the clinical population 0.79 for the OQ total score and for the three domains 0.76 (SD) 0.83 (IR), and 0.74 (SR). Internal consistency (Cronbach’s alpha) was 0.93 for the OQ total score and for the three domains 0.91 (SD) 0.80 (IR), and 0.69 (SR) [26]. In the cohort of the current study (*N* = 2,634) Cronbach’s alpha’s were similar; 0.93, 0.91, 0.79 and 0.66.

Data from each patient were included if there were at least two OQ-45.2 measurements, one at the start of treatment and one either at one of the 12-week assessments or the end-of-treatment assessment. If the measurement at the start of treatment was missing, the nearest measurement, for instance the measurement during intake, was used if the time between this measurement and the start of treatment did not exceed six weeks.

Positive outcome was defined in two ways, namely improvement or recovery. Improvement and recovery were based on the difference between the OQ-45.2 total score at the start of treatment and the OQ-45.2 total score at one of the 12-week assessments or at the end-of-treatment assessment. At the first moment during treatment when the difference score reached the criteria for improvement or recovery, the difference score was considered a positive outcome. The criteria for improvement and recovery were based on Jacobson and Truax’s criteria for reliable and clinically significant change [28]. To determine which change on a questionnaire score is an actual change and not just random fluctuation, Jacobson and Truax have proposed the Reliable Change Index (RCI). The RCI is calculated as the difference between the OQ-45.2 total score at the start of treatment and the OQ-45.2 total score at one of the 12-week assessments or at the end-of-treatment assessment, divided by the standard error of measurement. If the RCI is greater than 1.96, the change is considered statistically significant and the patient changes reliably. Furthermore, Jacobson and Truax defined clinical significant change as ‘the extent to which therapy moves someone outside the range of the dysfunctional population or within the range of the functional population.’ This requires a cut-off value for a specific instrument. The definition of recovery therefore, has more meaning and is somewhat ‘harder’, than the definition of improvement. Improvement could therefore be better described as ‘experiencing improvement’. However, for the readability in this article we only use improvement.

Improvement or reliable change on the OQ-45.2 was defined as a decrease by 14 points or more in the total score. Recovery was defined both as a decrease by 14 points or more in the total score and by having a total score less than the cut-off score for differentiation between the clinical and normal score ranges. The cut-off score for the Dutch version of the OQ-45.2 is ≤55 [27].

Because the measures were administered every 12 weeks, the speed of improvement or recovery was measured in an approximate way. It could appear in the analyses only every 12 weeks, or when treatment was terminated. This, however, was similar for all three of the treatment programs and for the different frequency categories.

### Potential confounders

Based on earlier mentioned potential predictors, adjusted analyses included the following variables: sex [6], age [7], functional impairment [7–9], severity of symptoms (OQ.45–2) at start of treatment [10] [9] [11] and group therapy [24]. Because all of these variables could have affected treatment outcome, they were included as covariates in the Cox proportional hazards model. Year of start of treatment, treatment program, and group therapy were also included because during the study period there were changes in the treatment protocols for the different diagnostic groups at the mental health care facility, and these changes could have affected treatment outcome. Group treatment was divided into a psychotherapy group or another kind of group, such as a supportive group or creative and psychomotor therapy. The number of available measurements was included because if a patient had many assessments, this might have increased the opportunity to detect improvement and recovery. Global Assessment of Functioning (GAF) at start of treatment was used as measure for functional impairment. The GAF scale was included in DSM-IV and rated for each patient at intake. Scores range from 100 (extremely high functioning) to 1 (severely impaired). Finally, medication could have influenced the number of sessions (pharmacotherapy contacts) as well as treatment outcome. Therefore, a variable ‘medication contact, yes/no’, based on the presence of pharmacotherapy contacts was also included as covariate.

### Analyses

We did not take patients’ outcome at the end of treatment into account, because it would not have distinguished between fast and slow responders to treatment. Instead, we used Kaplan-Meier survival analyses to identify the proportion of patients who had recovered at each month of treatment and Cox regression analyses to test the association between the number of sessions during the first phase of treatment and the potential confounders.

Data were analyzed using SPSS Version 20. To estimate the mean time to improvement and recovery of symptoms for the various session-frequency categories, Kaplan-Meier survival analyses were performed. Models were evaluated using the Chi-square of the log-rank (Mantel-Cox) test. Cox proportional-hazards regression models were used to estimate the hazard ratio for improvement of symptoms during treatment and recovery, according to the frequency of sessions during the first months of treatment. When patients did not improve or recover during treatment, their data were censored after the last assessment with the OQ45. We employed the enter method to include all of the covariates in one step in the Cox-proportional-hazard models. The comparison of models was based on the Wald statistic.

## Results

### Patients

Data from 2,634 patients were available for analysis. The characteristics of these patients are shown in Table 1. 62.8% (*n* = 1,653) of the sample were females, and their mean age was 36 years. 47.0% (*n* = 1,238) of the patients were offered treatment for an anxiety disorder; 35.0% (*n* = 922), treatment for a depressive disorder; and 18.0% (*n* = 474), treatment for a personality disorder (mainly personality disorder NAO, 65.1%, and cluster C, 30.6%). 29.4% of the patients had at least one pharmacotherapy contact. The mean number of treatment sessions in the first three months was 9.3 (*sd* = 7.7) 26.3% of the sample received group therapy in addition to individual therapy. The mean number of available measurements with the OQ-45.2 for each patient was 4.2. The mean total score on the OQ-45.2 at the start of the treatment was 87.4 (*sd* = 23.4) (high/very high), and the mean GAF score was 54.9 (*sd* = 8.1) (moderate symptoms or problems in functioning). At the final assessment, 24.6% of the patients had recovered, and 43.6% showed a clinically significant improvement in symptoms, social role, and interpersonal relationships. In these 43.6% the recovered patients were included.

### Kaplan-Meier survival analyses

The Kaplan-Meier survival analyses showed differences in survival distributions for improvement (log-rank Chi-square = 28.2, *p* < 0.001) and recovery (log-rank Chi-square = 28.8, *p* < 0.001) according to session frequency in the first three months. Because of the possibility of differences between the frequency categories on baseline measurements, Cox’s proportional-hazards regression was used to control for potentially confounding factors.

### Cox’s proportional-hazards regression analysis

Table 2 shows the results of the Cox’s proportional-hazards regression analysis. Compared to the group with 1–3 sessions in the first three months, the hazard ratios for improvement varied from 1.28 (95% CI = 1.08–1.53) for the group with 4 to 6 sessions to 1.62 (95% CI = 1.35–1.95) for the group with more than 12 sessions in the first three months of treatment. Hazard ratios for recovery ranged from 1.46 (95% CI = 1.11–1.92) to 2.04(95% CI = 1.53–2.72) for the groups with an increasing frequency of sessions during the first three months of treatment.

**Table 2:** Adjusted hazards ratios for recovery and Improvement (Cox regression analysis)^1^

Characteristic	Categories	Improvement	Recovery
Number of sessions in the first 3 months	1–3 (ref)^2^		
	4–6	1.28 (1.08–1.53)	1.46 (1.11–1.92)
	7–9	1.30 (1.06–1.59)	1.32 (0.96–1.83)
	10–12	1.52 (1.28–1.81)	1.72 (1.31–2.26)
	> 12	1.62 (1.35–1.95)	2.04 (1.53–2.72)
Sex	Male (ref)		
	Female	1.01 (0.90–1.12)	1.05 (0.89–1.23)
Age in years		1.00 (1.00–1.01)	1.00 (0.99–1.00)
Year treatment started	2011 (ref)		
	2012	1.49 (1.26–1.76)	1.41 (1.11–1.80)
	2013	1.55 (1.31–1.83)	1.54 (1.20–1.97)
	2014	1.99 (1.65–2.41)	1.89 (1.42–2.52)
	2015	2.63 (1.98–3.49)	2.39 (1.54–3.70)
Diagnostic program	Anxiety (ref)		
	Depression	0.85 (0.76–0.96)	0.99 (0.82–1.19)
	Personality disorders	0.72 (0.61–0.85)	0.79 (0.62–1.02)
Pharmacotherapy contact	No (ref)		
	Yes	0.97 (0.86–1.09)	0.88 (0.73–1.05)
Number of available measurements		0.86 (0.84–0.88)	0.89 (0.86–0.92)
Group therapy	No group therapy (ref)		
	Psychotherapy group	0.85 (0.68–1.06)	0.94 (0.67–1.31)
	Another kind of group	0.80 (0.70–0.92)	0.78 (0.63–0.96)
OQ-45 total score at start		1.01 (1.01–1.01)	0.99 (0.99–0.99)
GAF score at start		1.01 (1.01–1.02)	1.02 (1.01–1.03)

^1^Data are presented as hazards ratios with corresponding 95% confidence intervals, adjusted for age, sex, year treatment started, diagnostic program, presence of medication contacts, total number of OQ45 measurements, group therapy, OQ45 and GAF scores at the start of treatment

^2^Ref = reference category

Other independent variables that were associated with improvement and recovery were the year in which the treatment was started, the number of available measurements, having received group therapy in groups other than psychotherapy groups, and OQ-45.2 and GAF baseline scores. Mean baseline severity (OQ-45.2) differed between the frequency groups, but there was no interaction effect on outcome. Having pharmacotherapy contacts was not associated with improvement or recovery. The kind of diagnosis-specific treatment was associated only with improvement and not with recovery. The chance of improvement in the treatment programs was lower for patients with depression or a personality disorder than in the treatment program for patients with an anxiety disorder, obsessive-compulsive disorder, or post-traumatic stress disorder. Nevertheless, adjustments for these variables did not change the direction of the hazard ratios for session frequency. Session frequency in the first three months of treatment remained a significant predictor of treatment outcome, as shown by the adjusted hazard ratios in Table 2.

**Fig. 1:**
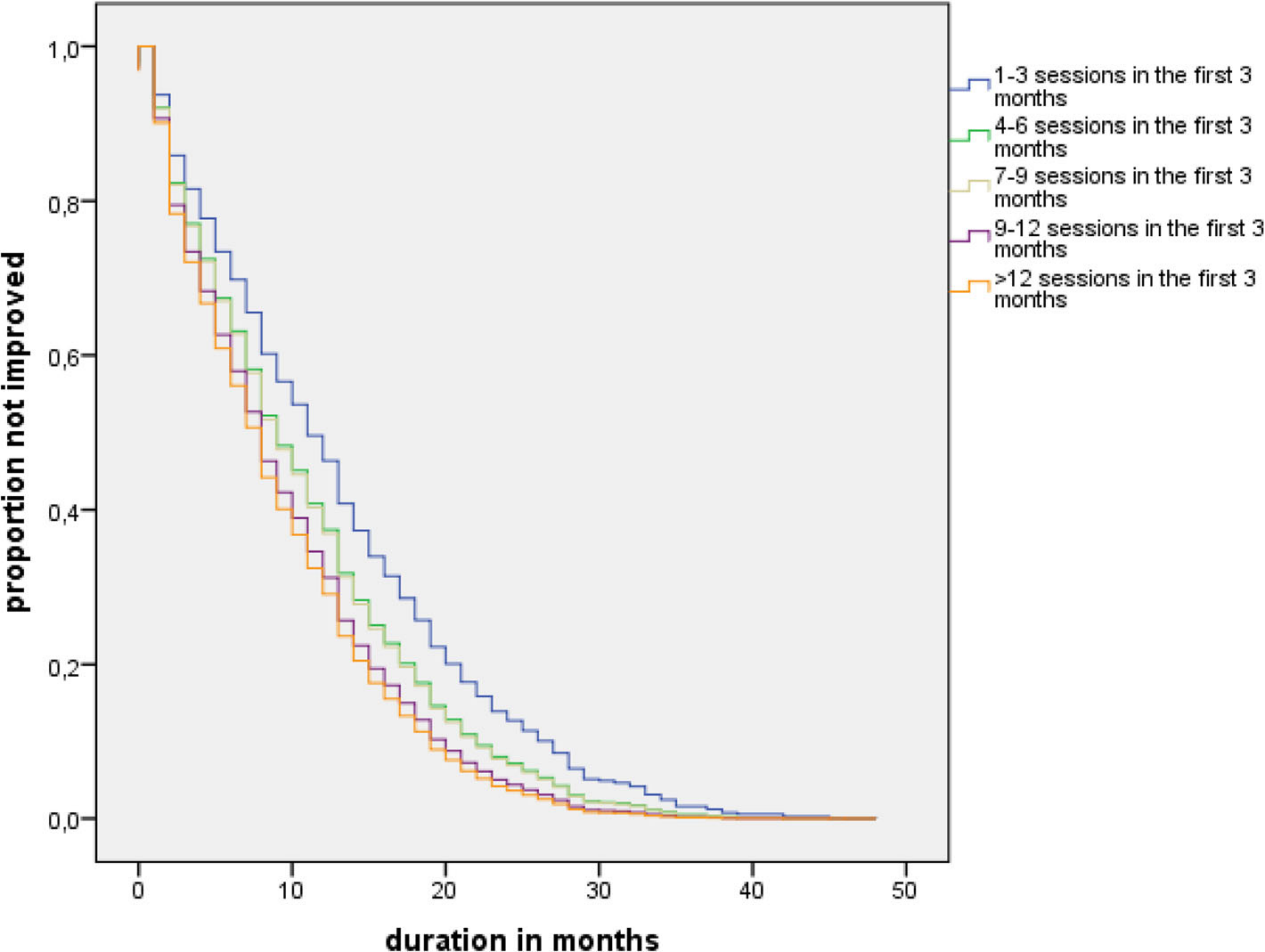
Survival function for improvement according to session frequency categories

**Fig. 2:**
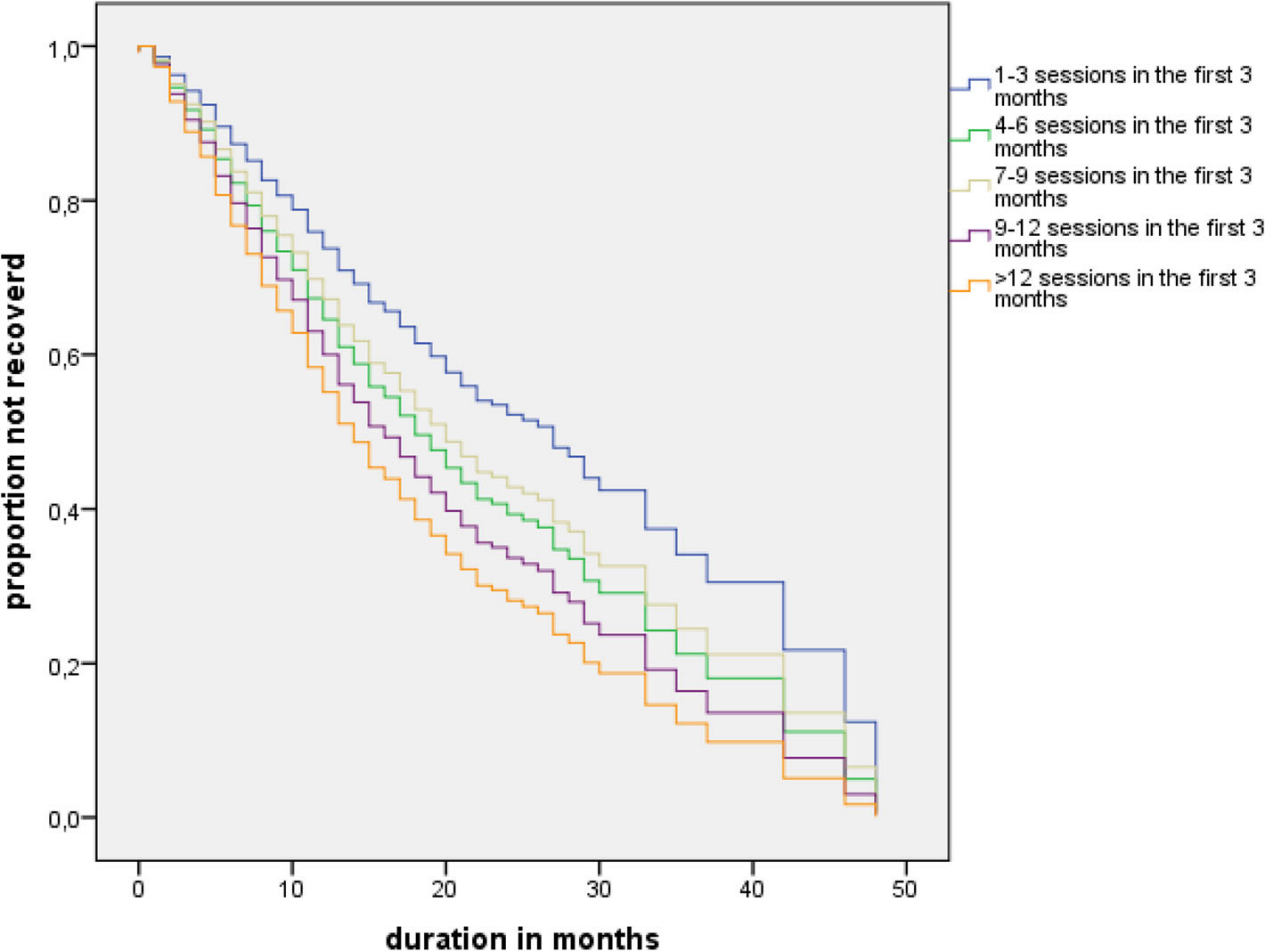
Survival function for recovery according to the session frequency categories

Figures 1 and 2 show the survival functions based on the adjusted hazard function of the Cox proportional hazard model. The results were independent of the diagnostic group, similar patterns were found in the survival curves for the individual three treatment programs. The interactions between frequency groups and diagnostic groups were neither significant for improvement (*p* = 0.61) nor for recovery (*p* = 0.82).

Because a longer episode increases the risk of chronicity [29], the risk after 12 months treatment can be interpreted as an indication for treatment resistance or chronicity. In the different categories of session frequency, we fitted stratified Cox models to the strata function for each category. The estimated baseline hazard functions for each stratum allowed us to compare the hazard proportions for improvement and recovery of patients after 12 months of treatment (see Table 3). From the lowest to the highest frequency category, the proportion of improved patients increased from 50.3 to 75.2% and the proportion of recovered patients increased from 26.6 to 45.9%.

**Table 3:** Hazard proportion improvement and recovery after 12 months, stratified by frequency level

Session frequency in the first 3 months	Improvement	Recovery
1–3	50.3%	26.6%
4–6	60.3%	34.5%
7–9	63.2%	34.1%
9–12	69.7%	38.9%
> 12	75.2%	45.9%

## Discussion

The aim of this study was to assess whether there is an association between frequency of sessions in the first three months of treatment and speed of recovery, and whether the association is the same for patients with depression, an anxiety disorder, or a personality disorder. Two outcome measures were used: (a) improvement, which was defined as a reliable, significant change, and (b) recovery, which was defined as a reliable, clinically significant change. Both outcome measures were associated with frequency of treatment sessions in the first three months of treatment.

Patients improved or recovered faster if their treatment was provided in a higher frequency of sessions during the first three months as compared to a lower frequency of treatment sessions. After one year, 25% more patients had improved in the highest frequency group than in the lowest frequency group, and 20% more patients had recovered in the former group than in the latter. After three years, in the lowest frequency group, as compared to the higher frequency groups, a substantially larger proportion of the patients had not recovered and were still in treatment. These results provide an initial indication that a low frequency of sessions at the beginning of treatment might lead to a more chronic course of the disorder in a substantial proportion of patients. Moreover, this pattern was similar in the three different diagnostic groups.

Although the association between session frequency and outcome was the same in the three diagnostic groups, the chance of improvement was lower in the programs for depression and personality disorder than in the treatment program for patients with an anxiety disorder, obsessive-compulsive disorder, or post-traumatic stress disorder. This is in line with other studies that show larger effect sizes in patients with anxiety disorders than in patients with depression for disorder specific interventions, but not for transdiagnostic psychological treatments [30], and in line with studies that show that having a comorbid personality disorder has a worse effect on treatment outcome [6, 8, 10].

As expected severity at start of treatment as measured with the OQ-45.2 and the GAF scores were related with improvement and recovery. In accordance with other studies [7–9] less functional impairment as measured with the GAF, increased the chance of improvement and recovery, but this was different for the OQ-45.2. The higher the OQ-45.2 score at start the higher the chance of improvement, but the lower the chance of recovery. As Hiller et al. [31] stated, the disadvantage of the RCI (the definition of improvement) is that it is independent of the initial score. Patients with higher initial scores in general tend to achieve larger improvements than patients with lower scores and therefore reach more easy a reliable change. The clinical significant change (recovery) does not have this disadvantage, because for recovery the score has to decrease until the cutoff between clinical and normal scores.

Some treatment variables were associated with improvement and recovery. The chance of improvement and recovery increased with each following year in which the treatment started. This probably reflects the process of continuous improvement of the treatment programs during these years. An alternative explanation might be that in the more recent years patients with a more chronic course of their disorder were not yet included. Participating in group treatment was another variable associated with outcome. In this study group treatments were divided into group psychotherapy and other kind of groups, such as a supportive group, art therapy or psychomotor therapy. Omar et al. found better outcomes among patients with borderline personality disorder who received more than one individual session per week or when patients received group-based sessions [23]. In the present study patients receiving care in the other groups (not group psychotherapy) had a lower chance of improvement and recovery. These kind of supportive, creative and psychomotor therapy groups are mainly provided as add-on therapy to patients with severe mental illness, which may explain this less favorable course. The number of available measurements was included in the analyses because if a patient had more assessments, this might have increased the opportunity to detect improvement and recovery. However, the opposite was found. The more measurements the lower the chance of improvement and recovery. More measurements also relate to a longer treatment course and therefore to a later moment of improvement and recovery.

The results in the current study corroborate earlier findings for depressive disorders [21], anxiety disorders [22], and borderline personality disorders [24]. Other naturalistic studies with heterogeneous samples showed similar results. However, these studies were conducted mainly with student populations [23, 32]. Most studies focused on frequency of sessions during the entire course of therapy rather than the first three months. Only Kraft et al.’s study [33] investigated the impact of session frequency in the first three months, as in our study. They found that the distribution of sessions during the first three months was similar for the three modalities of psychotherapy (psychodynamic, cognitive behavioral, and psychoanalytic) that they assessed, and it was not related to baseline severity. Although patients with fewer *sessions* in the first three months of psychoanalytic therapy improved somewhat more than patients with more sessions, patients who had fewer *weeks* with psychotherapy in the early phase of treatment subsequently improved at a slower rate. Patients showed better outcomes when their treatment started continuously, that is few weeks without psychotherapy, but at a rather slow pace. This finding was not seen in the other two forms of psychotherapy, where frequency or weeks without psychotherapy did not predict subsequent improvement.

There are several explanations for the benefit of starting treatment at a higher dose. Bruijniks et al., for instance, hypothesize two mechanisms of change [34]. First, a faster start of treatment may be associated with a faster development of the therapeutic alliance, which, in turn, is positively associated with treatment outcome [35]. The second mechanism refers to the learning process. Higher session frequency may increase the learning process, so that patients are better able to recall the content of the sessions and apply it in their everyday lives. In addition, Erekson et al. hypothesize, based on behavioral theory, that continuous reinforcement is most effective for learning new behavior, and longer gaps between treatment sessions lead to a discontinuity in learning [23]. The research underlying Kluger and DeNisi’s feedback intervention theory confirms that frequent messages augment the effect of feedback on learning [36].

The practical implications of the present study are important: to prevent a less favorable or a chronic course it is crucial to start the treatment not long after admission and to keep the frequency of the treatment sessions high. Therefore, it is first necessary to instruct both patients and therapists about the importance of a treatment intensity of at least one session per week during the first three months. Second, also managers should be instructed. As decreasing treatment intensity and session frequency is often used by managers to increase the caseload per therapist and to solve waiting lists, the current study seems to indicate that the opposite is true. Doing so will increase treatment time and a more chronic course in patients and will in fact lead to both increasing treatment time and increasing waiting lists (it takes more time to reach improvement).

Both the strengths and limitations of this study are related to its naturalistic design in a real-life mental health care facility. One strength is that this naturalistic nature of the design and setting increased the ecological validity and generalizability of the results. A second strength is that we were able to utilize data from highly prevalent diagnostic categories in large numbers. This allowed us to explore the association between session frequency and outcome and the association between session frequency and risk of a chronic course in patients suffering from depression, an anxiety disorder, or a personality disorder.

The major limitation of this study was the lack of experimental controls in the design. The patients, for example, were not randomly assigned to the different frequency groups. Although we were able to control for potential confounders, such as patient characteristics, which might have been associated with session frequency and treatment outcome, we could not control for assessment procedures, nor therapists’ selective allocation of patients to treatments. Moreover, it was impossible to control for all of the potentially confounding patient characteristics, such as duration of symptoms at the start of treatment. If the treatment of patients with more chronic problems had been systematically started at a lower frequency, chronicity might have been a stronger predictor of a chronic course than a low frequency of sessions at the onset of treatment. Furthermore, a low frequency of treatment sessions is often caused by practical or organizational problems, such as, a vacation or sick leave of a patient or therapist, waiting lists, and organizational difficulties. Because it was impossible to control for these variables, we do not know whether they were randomly distributed or more common in certain programs or treatment teams.

We also were unable to control for patients not attending their scheduled appointments (no-show) as a cause for low frequency of treatment sessions. Defife and colleagues [37] identified four groups of reasons for missed appointments: either medical or psychiatric clinical problems (28%); practical difficulties, such as work conflicts, transportation problems, and family issues (26%); motivational issues (18%); and adverse treatment responses, such as difficulties with the therapist or a negative response to the diagnosis or the recommended treatment plan (13%). Additionally, Murphy and colleagues [38] found that concerns about self-disclosure were related to nonattendance of the first appointment. A positive attitude toward therapy predicted increased rates of attendance among less depressed patients, but not among patients with severe depression.

Another limitation of the study is that assessments occurred only every 12 weeks. Because patients often completed the OQ-45.2 somewhat earlier or later than the scheduled time, the survival curves do not show events exactly every 12 weeks. This lack of uniformity might have affected the Kaplan-Meier curves. Nevertheless, we have no reason to believe that this effect differed among the five categories of session frequency. Because the number of assessments differed among patients and might have been associated with the occurrence of an event, we controlled for the number of assessments in the Cox regression analysis. Doing so, however, did not change the association between session frequency in the first three months and the speed of improvement or recovery.

## Conclusions

The mechanisms discussed above can account for the finding that a slow start of treatment is associated with slow improvement in the first phase of treatment. However, the results of the present study suggest that a slow start of treatment also affects the course of treatment in the long term and might increase the risk of treatment resistance, a persistent course of symptoms and poor prognosis. The clinical implications of this finding seem obvious. A quick start of treatment and adequate frequency of sessions in the initial phase of treatment for patients with a depressive disorder, an anxiety disorders, or a personality disorder may not only decrease patients’ symptoms and suffering faster, but it may also reduce the length of treatment and health care costs and can help to resolve waiting lists. Studies with more rigorous research designs are needed to confirm these hypotheses.

## Data Availability

The dataset used and/or analysed during the current study is available from the corresponding author on reasonable request.

## Abbreviations

CI: Confidence Interval
DSM-IV: Diagnostic and Statistical Manual of Mental Disorders 4th edition
GAF: Global assessment of functioning
IR: Interpersonal relations
M.I.N.I. 5.0.0: Mini-International Neuropsychiatric Interview version 5.0.0
OQ-45.2: Outcome Questionnaire-45
RCI: Reliable change index
RCT: Randomized controlled trial
ref.: Reference category
ROM: Routine outcome monitoring
SCID-II: Structured Clinical Interview for DSM-IV Axis II Personality Disorders
sd: Standard deviation
SD: Symptom distress
SR: Social role
